# Development of a Novel Design of Microfluidic Impedance Cytometry for Improved Sensitivity and Cell Identification

**DOI:** 10.1021/acsomega.3c00797

**Published:** 2023-05-16

**Authors:** Michael A. Warren, Amir Shakouri, Víctor Pacheco-Peña, Toby Hallam

**Affiliations:** † School of Mathematics, Statistics and Physics, Newcastle University, Newcastle upon Tyne NE1 7RU, United Kingdom; ‡ School of Physical & Mathematical Sciences, Nanyang Technological University, 21 Nanyang Link, Singapore 637371, Singapore

## Abstract

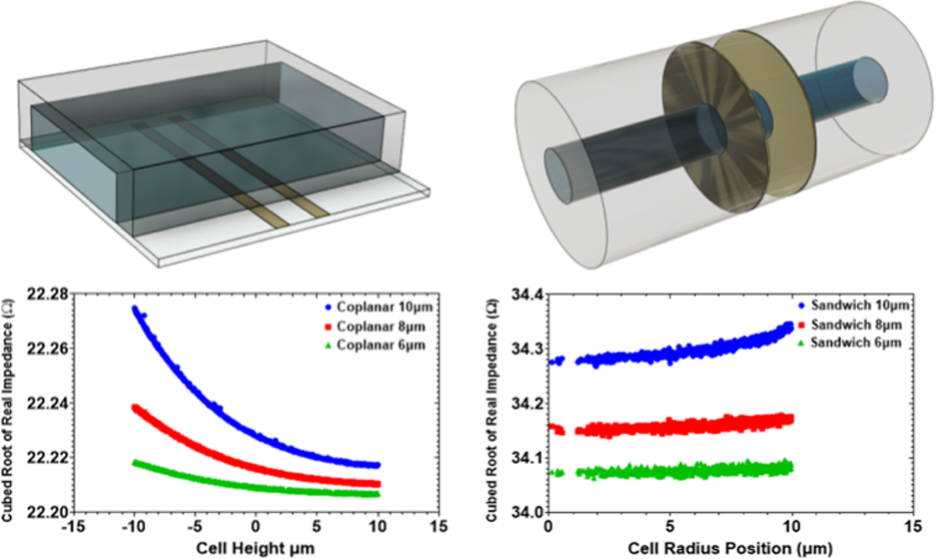

A long-standing issue for microfluidic impedance cytometry devices is the accuracy in determining the size of cells during counting and measurements. In this paper, we introduce a novel design that produces a homogeneous electric field in the sensing region and demonstrates higher accuracy than traditional designs in cell counting and sizing, reducing the reliance on cell focusing and signal postprocessing. The concept is validated, and the increased accuracy of the device over traditional designs is demonstrated through the use of finite element simulations to generate suitable data sets for particle trajectories and model expected signal variations.

## Introduction

In the field of biological measurements, techniques that allow label-free single-cell analysis have increasingly come into focus as they hold the promise of providing information that would otherwise be unavailable in bulk measurement methods.^1^ Microfluidic impedance cytometry (MIC) has been developed as a method that is capable of carrying out rapid, single-cell measurements in order to allow diagnostic tests to be carried out in challenging or resource-scarce environments such as conflict regions or developing countries.^2^ Disposable microfluidic cartridges and built-in signal conditioning equipment can carry out these measurements without the use of a static lab or ancillary equipment.^3^ Such approaches hold the potential for small, portable devices that can be used for other research applications including analysis of microorganisms,^1,4^ leukocytes,^5,6^ platelets,^7,8^ human cell lines,^9^ and plant cells, including the different steps in pollen development.^10^ These devices can also be utilized in industrial environments, such as in the testing of lubricant to verify standards^11^ or detect degradation on key machinery components as real-time condition monitoring devices.^12^ Such functionality can be key for equipment such as backup diesel engines or gas turbines which are often used on nuclear sites to protect essential supplies.^13^

The MIC technique makes use of alternating current (AC) excited electrodes embedded into a microfluidic channel that define a sensing region. Current flows between the electrodes, the amplitude of which varies, mostly due to the dielectric properties of the material within the sensing region.^14^ A sample containing particles or cells is suspended in a fluid solution which is then passed through the microfluidic channel; as each cell passes through the sensing region, a change in current can be measured due to the variation in material properties, i.e., electrical impedance, by measuring this signal variation, cell counting and sizing can be performed. Despite the obvious advantages of a microfluidic cell counting and sizing device, some key challenges remain that have prevented large-scale uptake such as the sensitivity of these devices to cell trajectory within the sensing region, which subsequently results in errors in the sizing of measured cells.^15^

The development of MIC devices has led to a strong focus on three main designs: (i) parallel electrodes,^14,16,17^ (ii) coplanar electrodes,^2,15,16,18,19^ and (iii) designs using a constriction channel.^7,20^ The most common of which are the parallel and coplanar electrode devices. One of the key issues of these devices is the inhomogeneity of the electric field in the sensing region, which can induce errors as cell position and size information is conflated. A number of studies have attempted to overcome the issue of nonhomogeneous electric fields in the sensing region through the use of novel designs and signal processing techniques;^1,5,7,21^ however, these modifications often result in complex manufacturing processes, heavy postprocessing, and in some cases, reduced sensitivity. A complete review of the basic principles of MIC devices and their development in the earlier years of research can be gathered from the review work by Cheung et al.^22^

In this study, we will explore a novel MIC design based on a sandwich of electrodes which addresses the weaknesses of the parallel and coplanar design by creating a homogeneous field distribution throughout the sensing region. It will be shown that this device can achieve a high sensitivity (defined as change percentage change in measured signal) while decreasing the induced error by offset cell trajectory in the sensing region, without the requirement for complex alignment procedures. The performance of this design is quantified by comparing this with the existing designs through studies involving numerical simulations. This is carried out without additional analysis techniques such as cell focusing^4^ and signal diagnostics,^19^ which is typically used to enhance the sensitivity and selectivity of these devices but can also reduce their effectiveness as a point-of-care device.

## Design and Modeling

In order to appropriately assess the performance of this novel device and provide a suitable comparison to the existing designs, appropriate models have been developed and in silico experiments have been performed using the commercial finite element software COMSOL Multiphysics.

**Figure 1:**
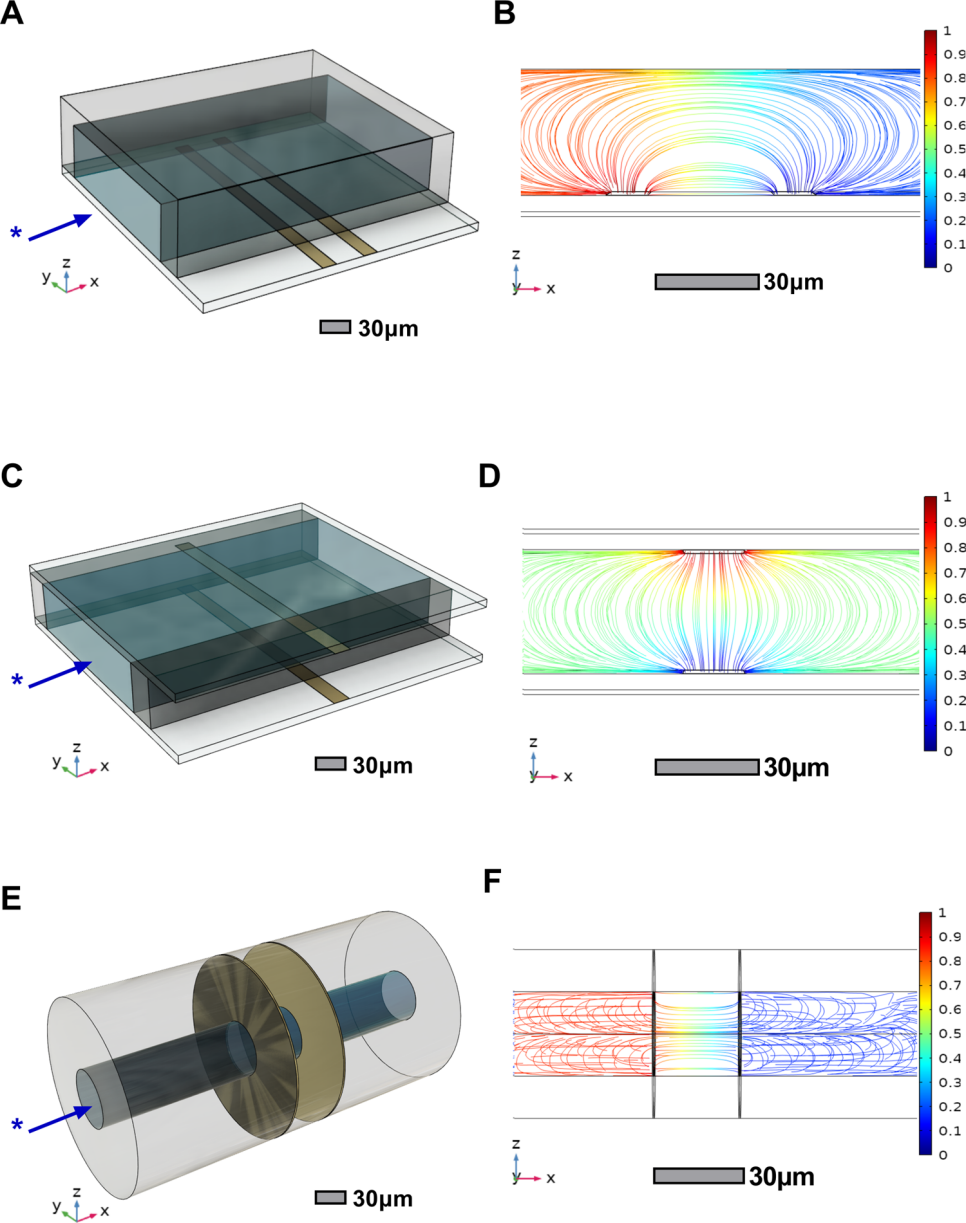
Geometry of the traditional MIC designs and a novel design examined in this paper. (A, C) Overall geometries of the coplanar and parallel devices, which have a microfluidic channel cross section of 30 μm height and 100 μm width. (E) Geometry of the proposed “sandwich” device, with a microfluidic channel diameter of 30 μm. (B, D, F) Electric field lines, as viewed from the *y*-axis of the coplanar, parallel, and novel sandwich design, respectively. The arrows marked * indicate the inlet fluid flow direction.

The geometries of the parallel, coplanar, and novel device design can be observed in Figure 1A,C,E, respectively. The first part of this paper will focus on the most basic form of these designs, utilizing only two electrodes and discussing the comparative performance of our novel design to those of the traditional designs. We will discuss more advanced designs of the novel device later in this paper.

We have modeled the variation in current flow between excitation and sensing electrodes due to the variation in conductivity and relative permittivity in the sensing region, based on steady-state calculations using a parametric sweep of the positions of the particle initially released at the center of the entry plane and traveling along a direct trajectory.

In order to model a representative biological cell passing through the devices, the cells have been modeled as spherical geometries within the device, while the cell membrane was modeled by a thin layer electric shielding condition, specified at all outer surfaces of the cell. This allows the electrical properties of the membrane to be specified for a representative single-shell cell model.^23^ Further details of the simulations, associated geometries, and their assigned material properties, including those of the suspending solution, are shown in the Supporting Information.

1 MHz was chosen for our simulations as an ideal frequency at which to measure cell sizing data due to the type of dielectric response expected from cells. Impedance measurements of cells suspended in solution are frequency-dependent and go through characteristic dispersions.^23,24^ At low frequencies (<100 kHz^24^), these devices are known to exhibit a largely capacitive effect due to the formation of an electric double layer (EDL) at the interface between the electrodes and the solution, which acts as a large source of capacitance in the measured signal, resulting in a large phase shift.^7^

The magnitude of the EDL can be reduced by operation of the device in sufficiently high frequencies; therefore, 1 MHz was selected as a suitable frequency to allow the magnitude of the EDL to be ignored, while avoiding the high-frequency cytoplasm polarization regions, which while rich in information is not related to cell size.^24^

In order to benchmark the novel sandwich design against those already prevalent in the literature, a finite element model for a coplanar, parallel, and novel design has been created and frequency domain analysis has been carried out at each desired position of the subject cell within the device. For these simulations, Dirichlet boundary conditions were implemented at the surface of the excitation electrodes, with an excitation voltage of 1 V AC at a frequency of 1 MHz. For the corresponding sensing electrode, a Dirichlet boundary condition was also implemented on its surface with a specified potential voltage of 0 V AC (ground). The resultant flow of current through the grounded sensing electrode is then measured, which varies as a particle or cell passes through the sensing region due to the variation in electrical conductivity and dielectric constant in the domain.

The excitation amplitude of 1 V has been selected following a review of previous works by Xie et al.,^20^ wherein it was determined that excitation amplitude should be maintained low to avoid cell damage. This excitation voltage is maintained throughout these studies in order to ensure results are consistent.

As reported in the literature, the sizing of the microfluidic channel and the sensing region (Figure 1B,D,F) is a compromise between throughput and the sensitivity of the devices,^2^ as this is governed by the dimensions of the sensing volume.^24^ Hence, cross-sectional dimensions of the microfluidic channel can be increased in order to reduce the chances of clogging but will suffer from a reduction in sensitivity. Due to this, it is recognized that each application will have individual design requirements which will be based on the diameter of the cells to be measured and may also depend on other cells present in the sample which require counting.

The dimensions of these devices were selected based on the potential measurement of whole blood cell counts; therefore, a minimum sensing region dimension of 30 μm has been specified in order to accommodate plasma, erythrocyte (red blood cells), leukocytes (white blood cells), and platelets^3^ with an additional margin for enlarged, cancerous cells.

To compare the particle sizing ability of these designs, a sample data set of cell trajectories for all designs has been created by using a simplified device geometry and application of the computational fluid dynamics module in order to analytically model the flow of phosphate buffer solution at a flow velocity of 400 μm/s through each device.

These equations were solved for the steady-state conditions present in a fully developed flow, following which the particle tracing module was then implemented in order to model the trajectory of particles through the device, based on random particle release positions at the inlet.

A freeze wall boundary condition was then specified centrally between the electrodes (at the point of highest sensitivity within the device) and cell positions captured after a data set of ∼706 cells was achieved. These cell positions have then been used within the parametric sweep studies using the stationary AC/DC solver in order to solve for the current flow within the devices at each cell position.

## Results and Discussion

In order to assess and compare the performance of the novel sandwich design against the existing conventional designs (coplanar and parallel), an analysis of the electric field line plots has been carried out to examine variations between designs. A sensitivity study was also carried out, to quantify the signal variation measured from the passing of a 10 μm cell through each device.

To ensure that the geometry of each device is fully optimized, parametric studies are carried out to determine the most sensitive designs to be modeled. Finally, a set of simulations to quantify the sizing accuracy of each device is carried out, using a data set obtained through fluid flow and particle tracing simulations.

### Electric Field Distributions

Figure 1A shows the physical structure of the coplanar device. Both electrodes are embedded into the bottom of the microfluidic channel, which is presented here with a representative cross section of 30 μm height and 100 μm width. These dimensions were chosen to reflect the experimental work being carried out on a coplanar device at the time of this work. It has been shown by Carminati et al.^2^ that the channel height dimension has the most significant effect on device sensitivity for the traditional designs, and therefore an increased width will have minimal effect on the accuracy of the device.

As it can be seen in Figure 1B, the electric field resulting from the electrode configuration in the coplanar device is nonhomogeneous and with a field intensity that decreases as one moves away from the electrodes. The field strength variation at varying heights introduces inaccuracy (blurring) in the determination of cell size as identical cells will produce different signals,^19^ depending upon the position within the microfluidic channel. This is one of the key disadvantages in this design and has led to the widespread uptake of complex strategies for signal conditioning^19,25^ and cell focusing^4,26^ in order to achieve suitable results.

An improvement in performance over the coplanar device is achieved for the parallel device shown in Figure 1C. Here, two electrodes are implemented, one at the top and one at the bottom of the microfluidic channel, again presented with representative dimensions of 30 μm height and 100 μm width. The electric field lines of the parallel device are shown in Figure 1D, and as the electrodes are located on opposite sides of the sensing region, the homogeneity of the electric field lines is increased over that of the coplanar device. However, there remains significant fringing of the electric field around the sensing region. It is also apparent that for electrodes with similar dimension to the coplanar device, the sensing region is reduced in size. The reduction in sensing volume results in a higher sensitivity, but the cost of this improvement is that during the manufacturing process, the electrodes must be precisely aligned when they are fabricated on either side of the microfluidic channel.^24^

An additional effect of reducing the sensing region in such a way is that larger cells, which extend outside of the small sensing region, will interact with the nonhomogeneous field and therefore reduce accuracy.

In this paper, we propose the structure shown in Figure 1E as an alternative device. As it is shown, the electrodes completely surround the microfluidic channel forming a sandwich structure that is embedded in the poly(dimethylsiloxane) (PDMS) matrix. Here, the channel has a circular 30 μm diameter channel throughout. Due to the electrode arrangement, the uniformity of the electric field is significantly improved (Figure 1F). The electrode edges extend away from the microfluidic channel, which removes the field fringing from the sensing region. This results in a much more homogeneous electric field distribution within the sensing region with excellent alignment of the electric field lines, through the sensing region. In the following studies, it will be shown that this alternative design results in high sensitivity and much greater cell sizing accuracy than that of the traditional coplanar and parallel devices. It will also be shown that the cell sizing accuracy of the novel device is much greater than that of the traditional designs.

### Sensitivity Study and Device Optimization

The electric field lines indicate the overall behavioral tendencies of the three comparative MIC designs shown in Figure 1. To quantify the difference in performance, we have carried out a comparative “single cell” sensing study in order to directly compare the relative current variation for each electrode configuration. In this simulation, each device is modeled as described in the design section above. The steady-state electric current is solved by using a stationary study of a single 10 μm cell at 100 points through the center of the sensing region to determine the maximum signal variation.

**Figure 2:**
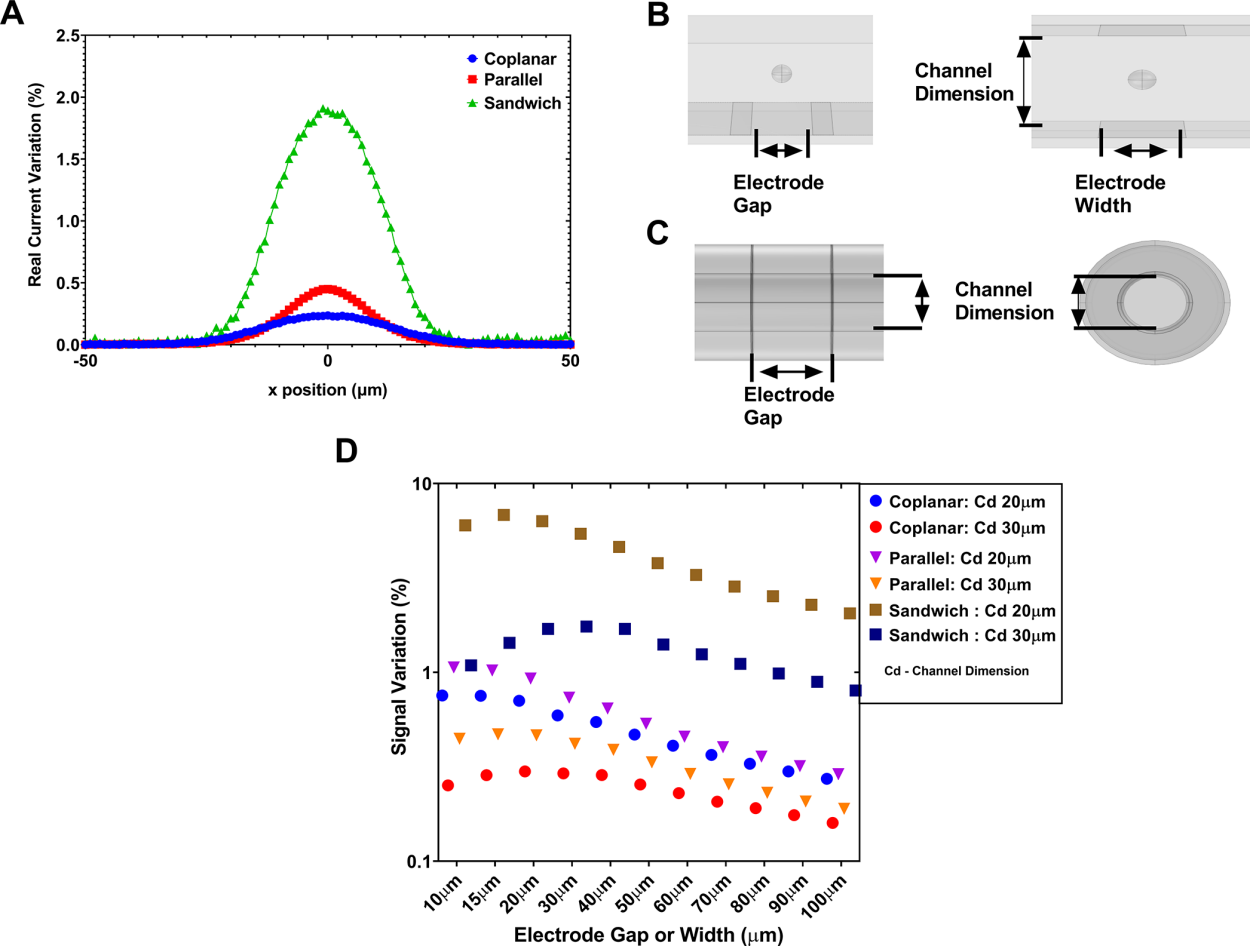
Two-electrode parametric design studies. (A) Sensitivity to a 10 μm spherocyte, (B, C) variable device dimensions with (D) associated design optimization results (note the log 10 scale of the *y* axis).

The results of this study are shown in Figure 2A, clearly demonstrating that the signal variation obtained by a cell passing through the sandwich device is larger than for the other two designs. It is important to highlight that the sensing volume is larger on the traditional designs, with an effective cross-sectional area of 3000 μm^2^ as opposed to 706 μm^2^ that is present in the proposed sandwich device. These measurements have been repeated for traditional device with more comparable dimensions, which demonstrates that the sandwich device still shows an increased sensitivity, this can be seen in the Supporting Information. For consistency, we relate these findings to those achieved by Cottet et al.^27^ who found a maximum signal variation of ∼0.35% for a coplanar device, in good agreement with our value of 0.29%.

To further explore the potential for increased sensitivity of these devices with the variation of key geometrical parameters (such as the channel height^2^), the effects of varying electrode gap and channel dimension were investigated using a parametric study (see Figure 2B,C). The geometry was defined using a set of global parameters for electrode gap and channel dimension, allowing the device geometry to be altered and the model re-meshed in order to solve for varying devices and cell positions.

The sensitivity was determined by modeling the presence of a cell both outside and in the most sensitive position of the device sensing region, so the maximum unit perturbation is gathered as the sensing signal in each case. Figure 2D demonstrates the sensitivity of all three designs to variations in the device geometry such as electrode gap (or width, in the case of the parallel design) and channel dimension.

It can be seen in Figure 2D that the sandwich design achieves a far greater sensitivity in all configurations, confirming that the increased uniformity in the electric field results in improved sensitivity. This may be of great benefit to device practicality due to a reduction in signal conditioning and analysis requirements in order to increase the signal-to-noise ratio.

The observed reduction in sensitivity as the electrode gap is reduced to the minimum value could be indicative of the current density being focused in the region immediately between the edges of the electrodes. This results in the electric field not fully permeating the central part of the sensing region, resulting in lower sensitivity. A similar effect is known as field compression which occurs when the channel height is reduced and has been discussed by Carminati et al.^2^

The electrode thickness for the sandwich device has been studied using the parametric sweep technique in order to evaluate the response of this parameter on this device. It was observed that this parameter had a very minor effect on the sensitivity of this device; however, it should be noted that due to the proposed fabrication technique, the electrode thickness would be expected to be of limited depth (additional details are available in the Supporting Information).

### Study of Cell Sizing

In addition to detecting cells, high-accuracy MIC should be able to unambiguously determine the size and type of the cell. Within existing designs, the challenge to obtaining accurate measurements of cell size is the variation of signal amplitude due to varying cell position in the channel.^15,24,28^ This issue arises from the inhomogeneity of the electric field within the sensing region of the device where the magnitude of the signal variation from a cell passing through the sensing region is dependent upon the position of the cell within the channel as well as the size of the cell.^14^ For example, in the coplanar design (Figure 1A), a small cell passing near the electrodes would show a similar signal variation to a larger cell traveling further away from the electrodes.

We have used a fluidic flow along with the particle tracing model from COMSOL Multiphysics to create a large (706 cells) set of particle trajectories through the sensing region of the three different electrode configurations, to represent a distribution of real cells in a sample flow through these devices. These simulations are key to obtaining representative results as there will be a significant variation in the passage of cells due to the cross-section velocity profile, for example, the higher-velocity region in the center of the microfluidic channel compared to the reduced velocity adjacent to the walls will result in more cells passing through this region, and therefore these should be appropriately represented.

This allows us to explore the interplay between cell size and position across the devices under study. Three different spherocyte cell sizes have been chosen (10, 8, and 6 μm) to be representative of blood cell species.^29^ These have then been used to calculate and illustrate the variation in signal due to cell distribution in the device. In order to ensure that cell geometry did not impact the results of this study, the geometry of all simulated cells (10, 8, and 6 μm) was specified as spherical to ensure only the cell size and position within the channel were varied.

**Figure 3:**
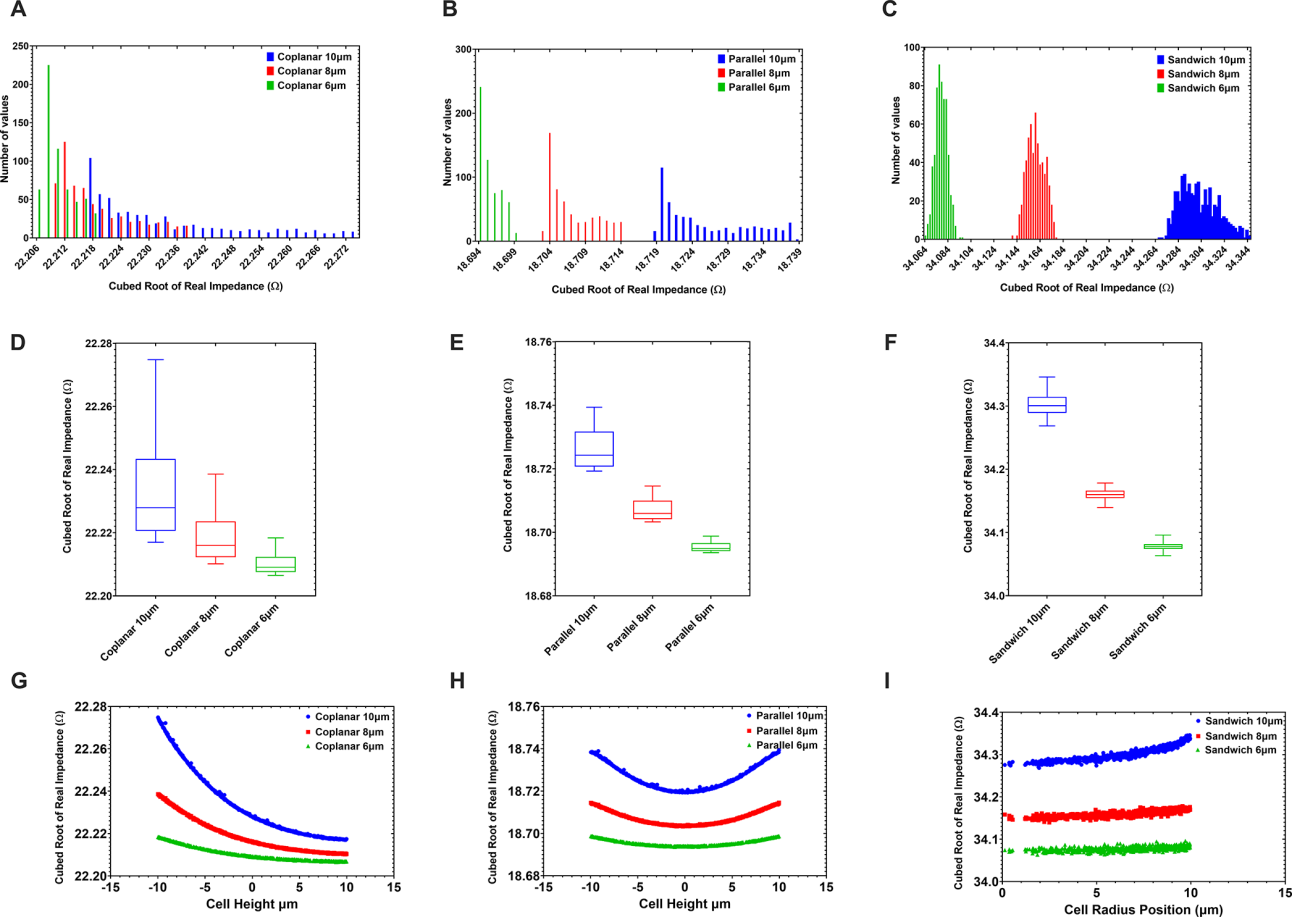
Two-electrode device accuracy study. (A–C) Histograms of impedance distributions of the measured responses of the coplanar, parallel, and sandwich device, respectively. (D–F) Box and whisker diagrams of the same data for the coplanar, parallel, and sandwich device, respectively, with the whiskers indicating minimum to maximum range of results. (G–I) Scatter plots which plot the cell height or radius position (for the sandwich device) against the impedance amplitude. These results illustrate the effect of the increased focusing of the electric field in the sensing region, which results in a clear reduction in the induced error of cell population size measurement (taken in cube root of impedance), based on variation of cell trajectory within the sensing region.

Each of the electrode designs were simulated for the sample of cells passing through, and the peak signal variations are presented as histograms in Figure 3. In an ideal case, the signal strength for each different cell size should be tightly clustered around a single value that scales linearly with cell volume. In the coplanar (Figure 3A,D) device where the field inhomogeneity is the largest, we can observe a considerable overlapping of measured values for differing cell sizes which makes cell sizing information almost impossible to extract from the raw data. For the parallel device (Figure 3B,E), the signal is more distinct, showing less overlap and the potential to differentiate between cell sizes with improved fidelity. Of concern is the large signal variation for the larger cells, which would make accurate sizing and detection of larger abnormal cells extremely difficult.

The sandwich design (Figure 3C,F) shows clear discrimination between the cell population size, with a large separation of the population’s signals, indicating an increased accuracy over both of the traditional designs.

In order to quantify the improved separation of cell size groups for the sandwich device, Brown–Forsythe analysis of variance (ANOVA) tests have been carried out to analyze the variance between measured groups of cell sizes for each device, while relaxing the assumption around equal variances across the data sets.

A result of particular note in ANOVA is the F ratio, which indicates the deviation from the null hypothesis (i.e., a value of 1 indicates that the data is sampled from data with the same mean; the higher the value, the more significantly separated the data, see eq 1).
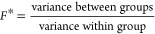
1The F* ratio is analogous to the *F* ratio for a standard ANOVA but with a variation in the calculation due to the use of the Brown–Forsythe ANOVA.

It can be seen from the results in Table 1 that the F* value for the coplanar device is the lowest of all designs, indicating the mean value for the population cell size shows much lower separation than for the sandwich design. This conflates to a greater error in cell sizing.

**Table 1:** Summary of ANOVA Tests for Cell Sizing in MIC Devices

device design	F* value
coplanar	758.8
parallel	5955
sandwich	64 655
three-electrode sandwich	116 802

These results give promise for the sandwich device to be used in order to size cell populations far more accurately than that of the traditional designs, reducing the requirement for additional cell focusing, the use of multifrequency opacity measurements,^25^ or novel signal differentiation techniques.^19^ Although these techniques have been proven to be effective, they may result in a more cumbersome device; here, we have been able to address these issues in the initial design stages, giving much higher accuracies through device geometry alone.

### Three-Electrode Design

**Figure 4:**
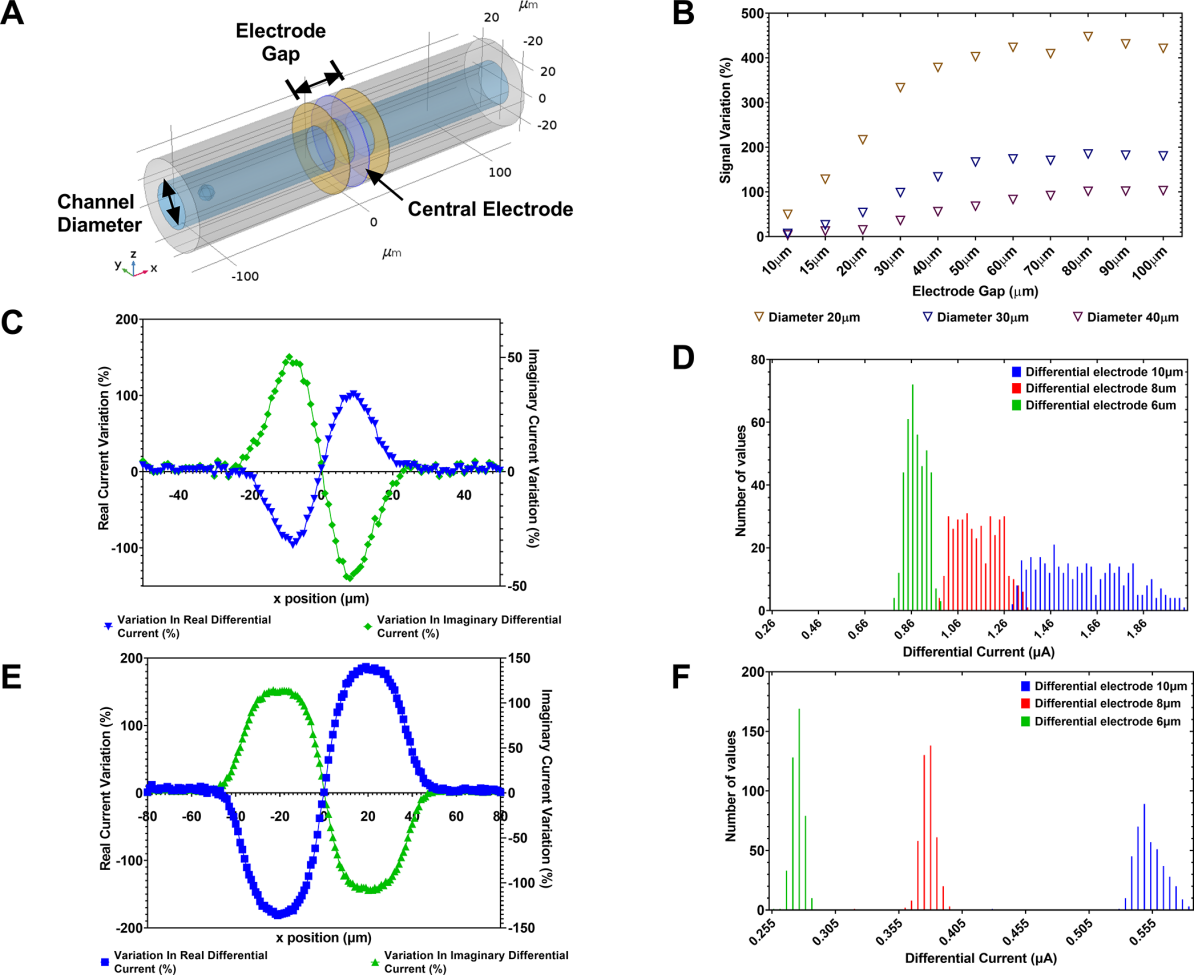
Grounded central electrode with differential current measurement. (A) Schematic representation of the proposed device showing the parameters that are adjusted during the studies. (B) Plot of device signal response throughout device optimization studies. (C) Initial variation in measured signal due to a 10 μm cell passing through the sensing region. (D) Histogram showing initial variation in measured signal due to cell position variation and (E) improvement in measured signal variation achieved by optimizing the device dimensions to an electrode separation of 80 μm and a channel diameter of 30 μm. (F) Histogram showing improved results for measured signal due to cell position variation, indicating the accuracy of the device has also been greatly improved, which can be seen by the greater separation of signal distributions for the sizes of cell groups (green, red, and blue histograms representing the three different cell groups, 6, 8, and 10 μm, respectively).

A potential application for extending the current model shown in Figure 1E could involve a third electrode, as is often the case with modern MIC devices. This may increase the sensitivity of the device by allowing a differential measurement to be carried out, with a signal of nominally zero when there are no cells passing through the sensing region.^2,24^Figure 4A shows the sandwich design in a three-electrode configuration where the central blue ground electrode is surrounded by two excited yellow electrodes. The electrodes to either side of the central electrode define two separate field regions.

This sets up a symmetric electric field on either side of the central grounded electrode, which results in a characteristic bipolar pulse (Figure 4C) being measured as a cell passes through the device. Compared to the two-electrode devices, these differential devices can yield higher sensitivity and the peak-to-peak time can be used to determine the cell velocity.^24^

In the study of this novel sandwich design, the two outer electrodes are excited at 1.0 V_ac_ 1 MHz, consistent with the earlier two-terminal device study. The central electrode is grounded, and the current passing through the excitation electrodes is evaluated. The resultant signal is measured as a differential signal (excitation electrode 1 real current–excitation electrode 2 real current) and results in a much higher sensitivity due to the use of this differential signal measurement.

The gap between the outermost electrodes is 30 μm; therefore, the reduction in the distance between the excitation and ground electrode results in a much higher gradient of electric field (6.53 × 10^5^ V/m in initial device configuration (associated data available in the Supporting Information)) compared to that of the two-electrode design (2.81 × 10^5^ V/m in optimized device configuration (associated data available in the Supporting Information)).

This then results in a highly sensitive response in the resultant trace of a cell moving centrally through the sensing region, which can be seen in Figure 4C. It should be noted that the sensitivity is defined as the percentile change in the measured differential current from the value when there is no cell present in the sensing region between the electrodes; therefore, a bipolar response is measured as a reflection of the signal moving through both sensing regions and effecting the measured signals independently.

It can also be observed in Figure 4D that the simulations carried out using a population of cells in a fluidic flow demonstrate excellent separation and therefore hold promise for this device to achieve high accuracy upon implementation. Analysis of variance testing was carried out on this data set, and an *F* value of 3584 and an *R*^2^ value of 0.8569 were achieved for these results, indicating that the groups (cell sizes) are significantly separated. These values are comparable to those found in Table 1 for the parallel device and show a poorer separation compared to that of the two-electrode sandwich device.

Using a similar method to the cell sizing study for the two-terminal device, a parametric study for the electrode gap and the channel diameter was then carried out to determine optimal dimensions for this differential sandwich design. Figure 4A indicates the device dimensions that were varied, where the position of the outer electrode separation changes and the central electrode is kept at the midpoint. The channel diameter is also varied between 20 and 40 μm. Figure 4B shows the signal variation obtained by comparing the signal for a cell outside of the sensing region and at the position within the channel which gives the highest response. As with the two-terminal device, the sandwich electrode configuration achieves its highest sensitivities with the lowest sensing volume (channel diameter of 20 μm).

In contrast, the electrode separations illustrated in Figure 4B do not follow this trend, and it is apparent that for smaller electrode gaps (<50 μm), the device exhibits lower sensitivity. This is due to the differential design having two sensing regions between outer excitation electrodes, resulting in an electrode gap of at least 60 μm required between the outer electrodes in order to achieve maximum sensitivity (30 μm per sensing region).

Taking the optimal electrode separation of 80 μm and a 30 μm channel diameter (which is desired in this case in order to allow whole blood cell counts^3^), the increased separation of the electrodes results in a significant increase in the sensitivity of the device, seen in Figure 4E, while excellent separation of cell sizing signals is observed in Figure 4F, indicating high accuracy of the device.

Brown–Forsythe analysis of variance testing was carried out on this data set, and an F* value of 116 802 was achieved for these results, again indicating that the groups (cell sizes) are significantly separated for the optimized, three-electrode device. This is a significant improvement over the two-electrode design. We have also carried out optimization for three-electrode devices in the coplanar configuration (the results can be found in the Supporting Information), and we find that the sandwich design is much more accurate for all designs.

## Conclusions

In this paper, we have described and evaluated the performance of a novel MIC device design against that of the equivalent traditional MIC device designs using the finite element method. The results of the simulation demonstrate that this device has an inherently higher sensitivity and is less susceptible to cell sizing measurement error due to variation of cell trajectories in the sensing region, which has been achieved by re-assessing the design from the ground up without the use of complex cell focusing or signal conditioning techniques.

The electrode configuration was investigated in both a two-electrode and three-electrode configuration in order to demonstrate how this variation in MIC design can be implemented in more advanced devices to yield higher sensitivities.

Beyond the improved performance, a significant benefit that should be highlighted is that of the device geometry, which would lend itself to simplified fabrication techniques (detailed further in the Supporting Information) rather than multistep layer-by-layer microfabrication methods with alignment techniques associated with existing designs.

Designs utilizing circumferential electrodes in the form of microtubes with internally wrapped electrodes have been explored in the literature; however, these involve much more novel microfabrication techniques than those described within this manuscript. Therefore, we believe that the techniques proposed in our manuscript may provide some groundwork to potential low-cost commercial production methods for MIC devices.

## Supporting Information Available

Computational simulation techniques and software used; description of the proposed fabrication techniques and supplementary discussion of results associated with the novel device; and further results for additional sensitivity studies carried out as part of this work (PDF)
