# Supplementary material for: Development of a Novel Design of Microfluidic Impedance Cytometry for Improved Sensitivity and Cell Identification
Source: ACS Omega. 2023 May 16;8(21):18882–90. doi: 10.1021/acsomega.3c00797 (PMC10233676; doi:10.1021/acsomega.3c00797)
Supplement: Supplementary file 1 — ao3c00797_si_001.pdf [file ao3c00797_si_001.pdf]

Development of a novel design of Microfluidic Impedance Cytometry for improved sensitivity and cell identification

Michael A. Warren*, Amir Shakouri, Víctor Pacheco-Peña, and Toby Hallam*

KEYWORDS: Cell Counting, Impedance Cytometry, Impedance Detection, Microfluidics

Scheme S1 - Supplementary Discussion for Design & Modelling

As the sensing mechanism for the MIC devices consists of a current flow through the sensing region which is affected by the dielectric properties within the domain, the associated AC/DC module has been implemented in order to solve Maxwell's equations for the electric field, current and potential distributions within the modelled domains.

Within the AC/DC Electric Currents module, the following current conservation equations (derived from the Maxwell equations) are solved for, in the frequency domain:

$$J = \sigma E + j\omega D + J_e \qquad \text{(Equation S1)}$$

Where J is current density (A/m^2), σ is the electrical conductivity (S/m), E is the electric field (V/m), ω is the angular frequency (rad/s), D is the electric displacement field (C/m^2) and J_e is an external current density source (A/m^2).

$$E = -\nabla V \qquad \text{(Equation S2)}$$

Where E is the electric field (V/m).

Quadratic tetrahedral elements have been adopted and the mesh designed to create a higher element density in regions of higher electric field gradient.

The meshing of the FEM model was carried out using a quadratic tetrahedral mesh in all domains and in order to ensure the mesh was sufficiently refined, mesh convergence studies were carried out until the readings converged to indicate minimal residual error.

The cell membrane has been modelled using a distributed impedance thin layer condition, although the difference in measurement was minimal between the approximated whole cell properties and the thin layer modelled properties, this will give a more accurate model of the cell while also reducing meshing requirements due to the variation in dimensions between the geometry and the thin membrane.
The PARDISO direct solver has been employed for these simulations, this was found to be the optimum solver as the iterative solvers employed were unable to suitably resolve the equations to the required level of residual error.

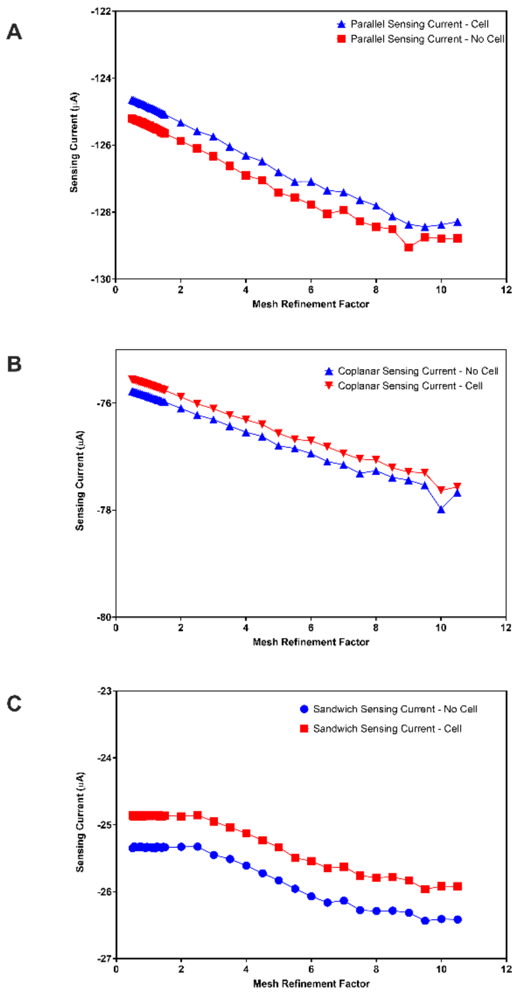

Figure S1 - Mesh convergence studies for the different designs discussed above were carried out in order to ensure that the level of refinement on the FEA mesh was sufficient to minimise the residual error. These studies have been carried out with both the cell centrally within the sensing region and outside of the sensing regions in order to confirm there was no divergence between these results.

Table S1 - Table of Modelled Material Properties for Electric Currents Simulations

Name	Expression	Description
sigPBS	1.6 [S/m][1]	Electrical Conductivity of PBS solution
sigRBC	0.2 [S/m]	Electrical Conductivity of cell interior
epsPBS	80[1]	Relative Permittivity of PBS solution
epsRBC	80	Relative Permittivity of cell interior
sigMem	0.0 [S/m] [2]	Electrical Conductivity of cell membrane
epsMem	10000 [2]	Relative Permittivity of cell membrane

Scheme S2 - Fluid flow with particle tracing simulations

To ensure accuracy of the CFD simulations, the mesh for these models utilised 8 boundary layers on all inner surface boundaries in order to more accurately resolve the fluid flow gradient at the no slip boundaries.

The inlet of the microfluidic device was defined as fully developed flow boundary condition with a flow rate of 400 μm/s while the boundary condition for particle release was specified at the inlet as a statistically random release boundary in order to provide a randomised data set of cells released into the fluid at regular periods.

A central 'capture' plane was defined in the most sensitive region of the device in order to place a freeze condition on the cells, once reached. This allowed a flow to be established, particles released, and cell co-ordinates obtained for passage through the point of peak sensitivity of the sensing region. A data set of cell positions could then be obtained and fed into the model which allowed parametric simulations to be run in order to create a data set based on this subset of particle positions.

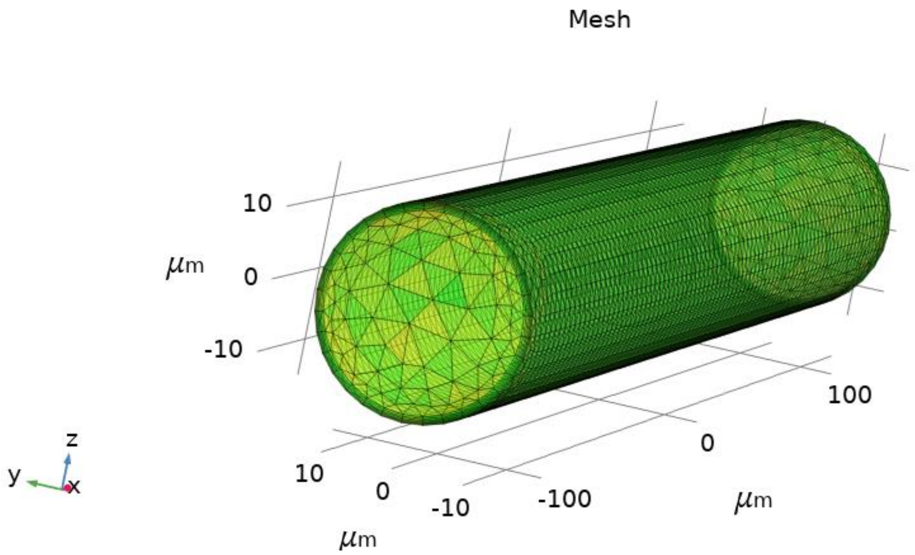

Figure S2 - 3D plot of meshing arrangement used for the modelling of microfluidic flow though the novel sandwich device. It can be seen that additional boundary layers have been applied at the walls of the microfluidic channel in order to suitable resolve the region of slip between the fluid and walls.

Scheme S3 - Proposed fabrication technique

It is proposed that the sandwich device could be manufactured through the use of a simple layer by layer technique, wherein PDMS is spin coated to the desired thickness onto a substrate before being cured [3]. A layer of gold electrodes are adhered onto the PDMS layer through sputtering deposition techniques, such as those discussed by Nag et al [4]. This process is then repeated to the desired number of electrodes, followed by a final spin coated layer of PDMS. The microfluidic channel could then be created through a process such as vibration assisted micro drilling, such as those methods reviewed by Hasan et al [5].

Although the geometry is modelled as a cylindrical device, this is to reduce the computational requirements in modelling sections of the device which have no significant effect on the performance of the device. There is no requirement for the device to have a cylindrical outer diameter, therefore the simplified method described could be employed to create such a device.

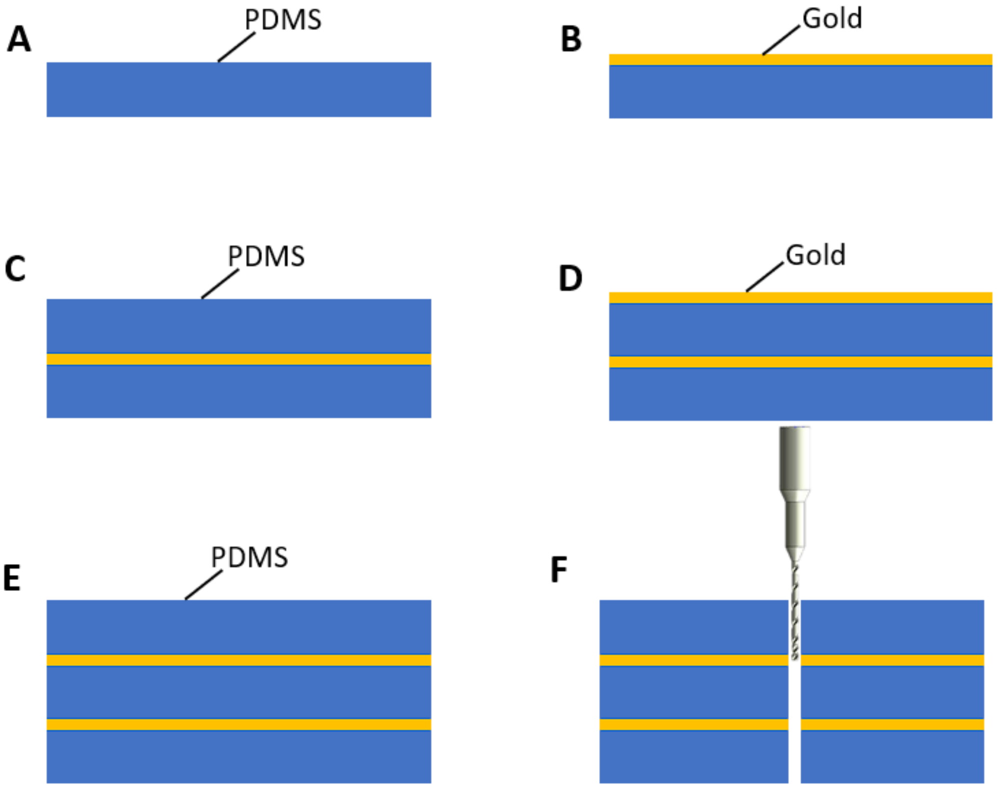

Figure S3 - Schematic diagram of proposed fabrication technique. A layer of Polydimethylsiloxane (PDMS) is deposited onto a substrate via spin coating to achieve the desired thickness (A). The electrode material is deposited onto the PDMS via sputter deposition of Gold (B). This process is then repeated to achieve the desired device configuration (C-E). Finally, micro-drilling technique is employed to create the microfluidic channel throughout the device (F).

Scheme S4 - Supplementary Discussion for Results

In order to ensure that the improved performance of the device was maintained with a comparable sensing volume between all three devices the Coplanar and Parallel devices were modelled with channel dimensions of 30μm x 30μm (W x H). The results shown in Fig. S4 below and the resultant ANOVA figures in table S2 give a clear indication of the improvement in performance of the sandwich device when compared to the traditional coplanar and parallel devices.

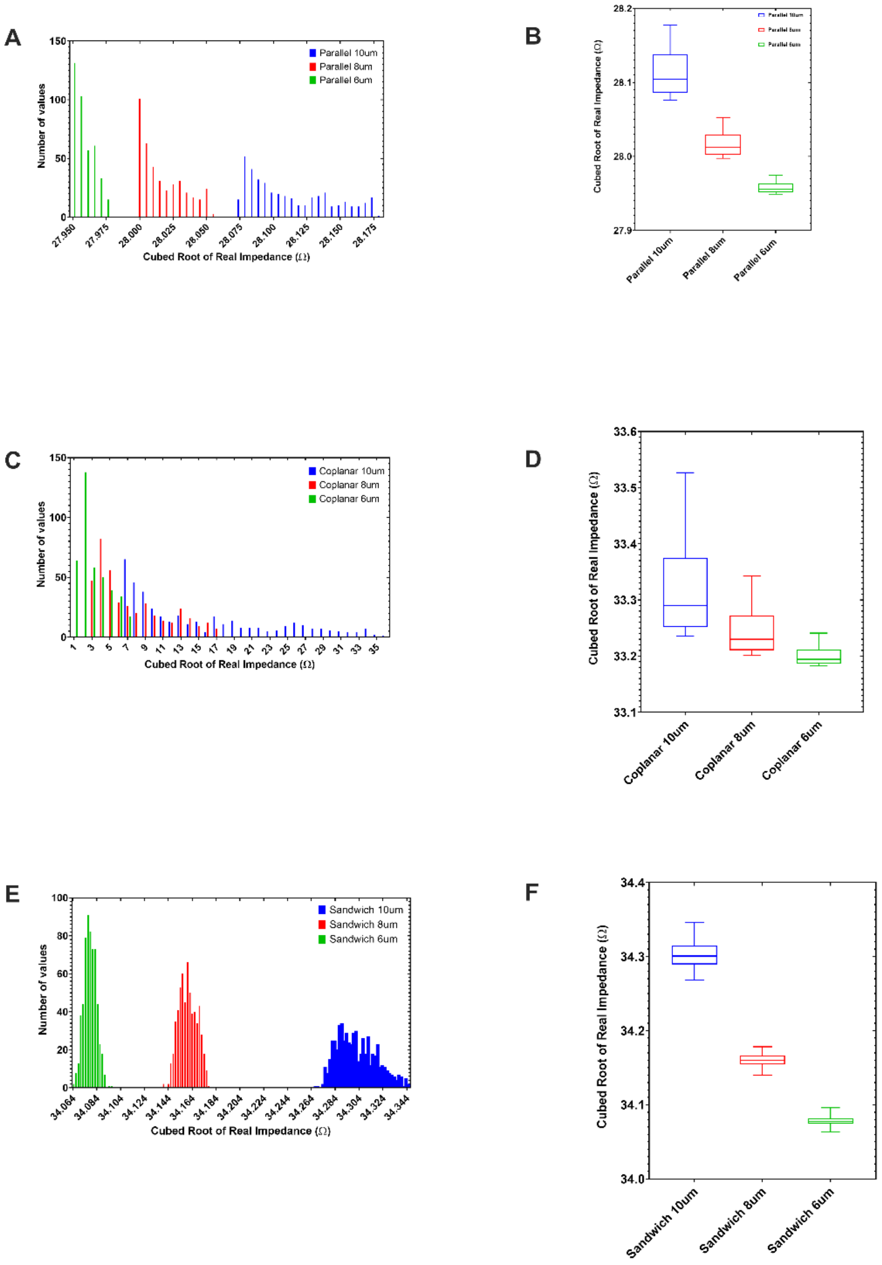

Figure S4 - These results illustrate the accuracy of each device, showing the effect of the increased focusing of the electric field in the sensing region of the novel device, which results in a clear reduction in the induced error of cell population size measurement (taken in cube root of impedance), based on variation of cell trajectory within the sensing region. (A, C and E) illustrates the histograms of impedance distributions of the measured responses of the parallel, coplanar and sandwich device, respectively. Box and whisker diagrams of the same data is displayed in (B, D and F), for the parallel, coplanar and sandwich device, respectively, with the whiskers indicating minimum to maximum range of results. This result shows that the accuracy is still greatly increased when the sensing volume of the traditional devices (Coplanar and Parallel) is reduced to that of the sandwich device.

Table S2 - Table of Brown-Forsythe ANOVA results for reduced sensing volume devices

Device Design	F* Value
Coplanar	5955
Parallel	528.1
Sandwich	64655

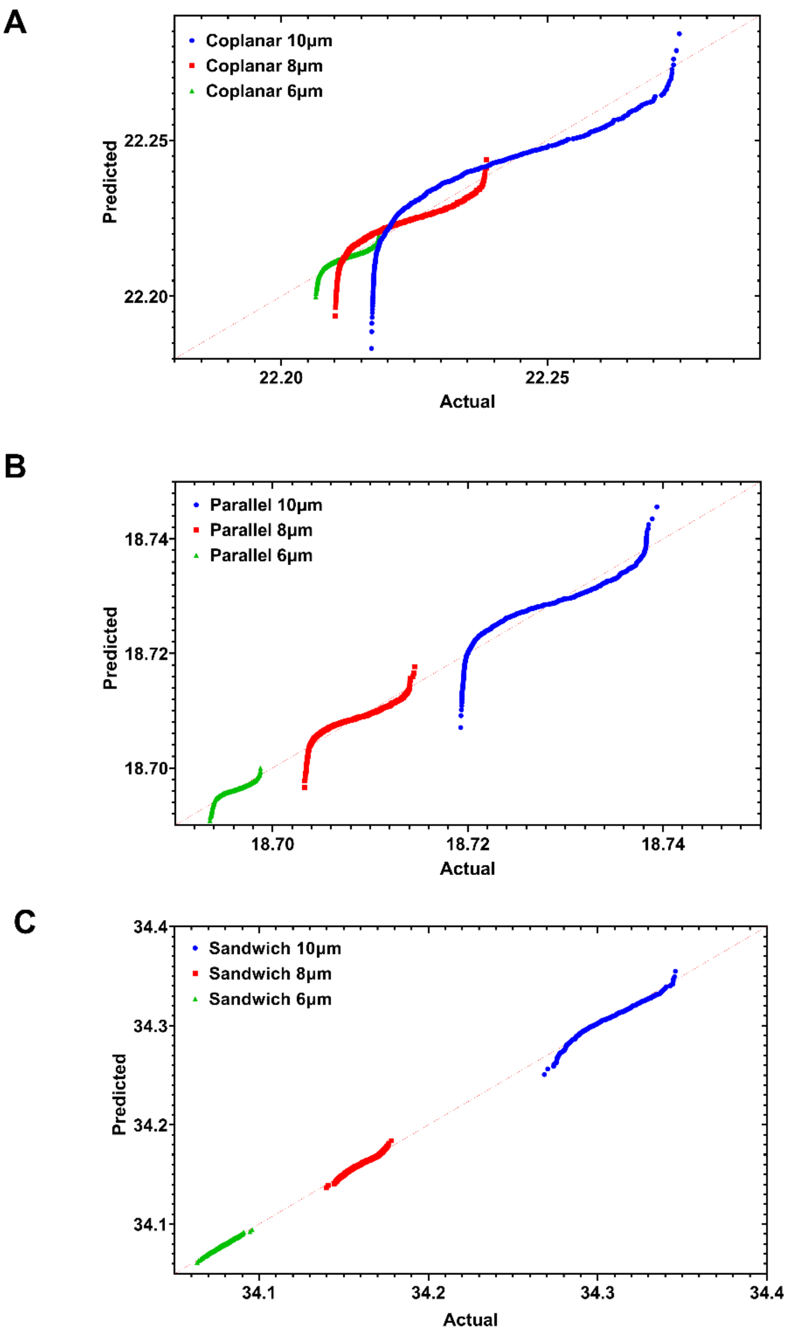

Figure S5 - Quartile-Quartile (QQ) plots of simulated RBC flow data for two electrode devices. (A) shows the results for the coplanar device, which shows a significant deviation from a normal distribution and over dispersion in these results. (B) shows that the parallel device shows a significant benefit in the distribution of results within the QQ plot. It should be noted that the QQ plot of the sandwich device (C) indicates that the obtained results follow a much more normal distribution than that of the coplanar and parallel devices, albeit with a slight increased tail on the 10μm results.

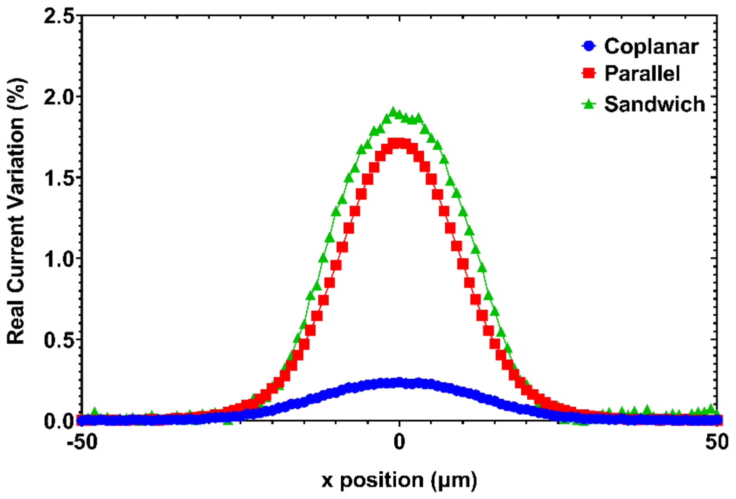

Figure S6 – Two-electrode device sensitivity plots for the sandwich device and traditional devices with reduced dimensions. The reduced dimensions result in a significant increase in sensitivity, particularly for the parallel device, however the novel sandwich design still shows a higher sensitivity than that of the parallel design.

A parametric study of the two-electrode sandwich device was also carried out in order to determine if there was a significant change in the device sensitivity when the thickness of the electrode is varied and response to a 10µm cell measured. The results of which are shown in Fig. S7.

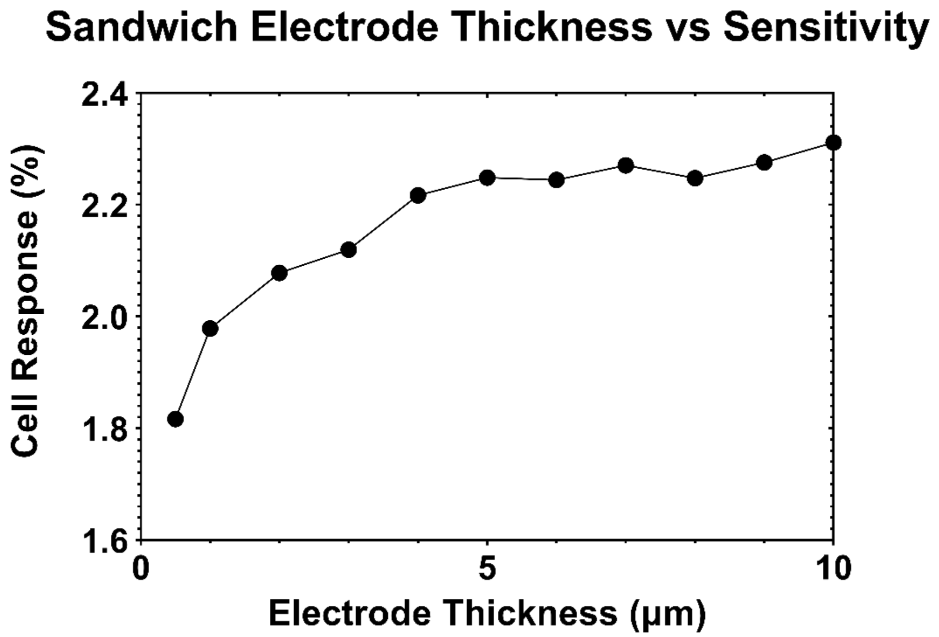

Figure S7 - Plot of two-electrode sandwich device response to a 10µm cell passing through devices of varying electrode thickness. It can be seen that there is a minor increase in the response sensitivity of the device as the thickness of the electrode is increased, which quickly begins to plateau at a thickness of 4 µm as the electrode thickness is no longer the limiting factor in current penetration into the sensing region. However, given the fabrication method described in Fig. S3 of the Additional SI, an electrode thickness greater than 0.5µm would not be desirable

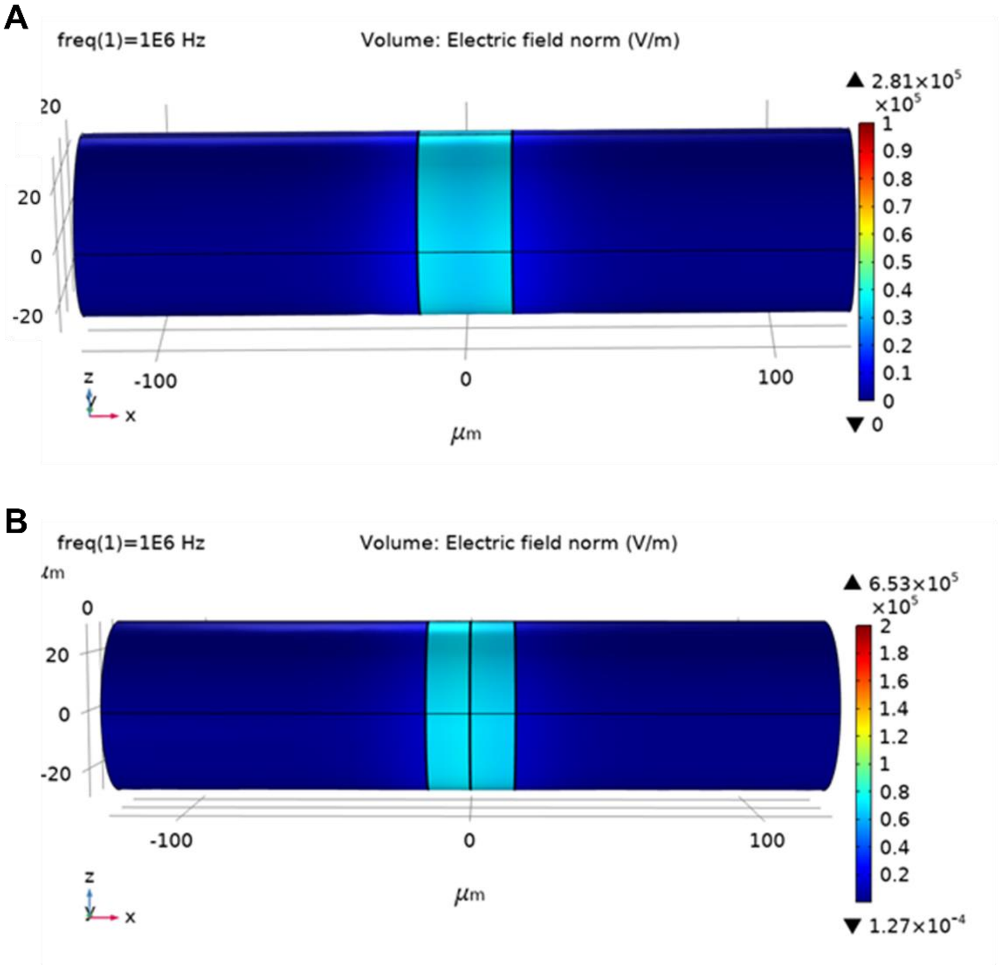

Figure S8 - Plots of normalised electric fields for the sandwich devices, maximum and minimum values of the electric field are indicated above and below the colour chart to the right of the image. (A) shows the normalised electric field of the two electrode sandwich device, while (B) shows that of the three electrode differential sandwich device. It should be noted that the outer electrode gap on both devices is 30 µm, therefore the addition of the central grounded electrode in (B) results in a larger gradient of electric field ($6.53x10^5$ V/m, compared to $2.81x10^5$ V/m).

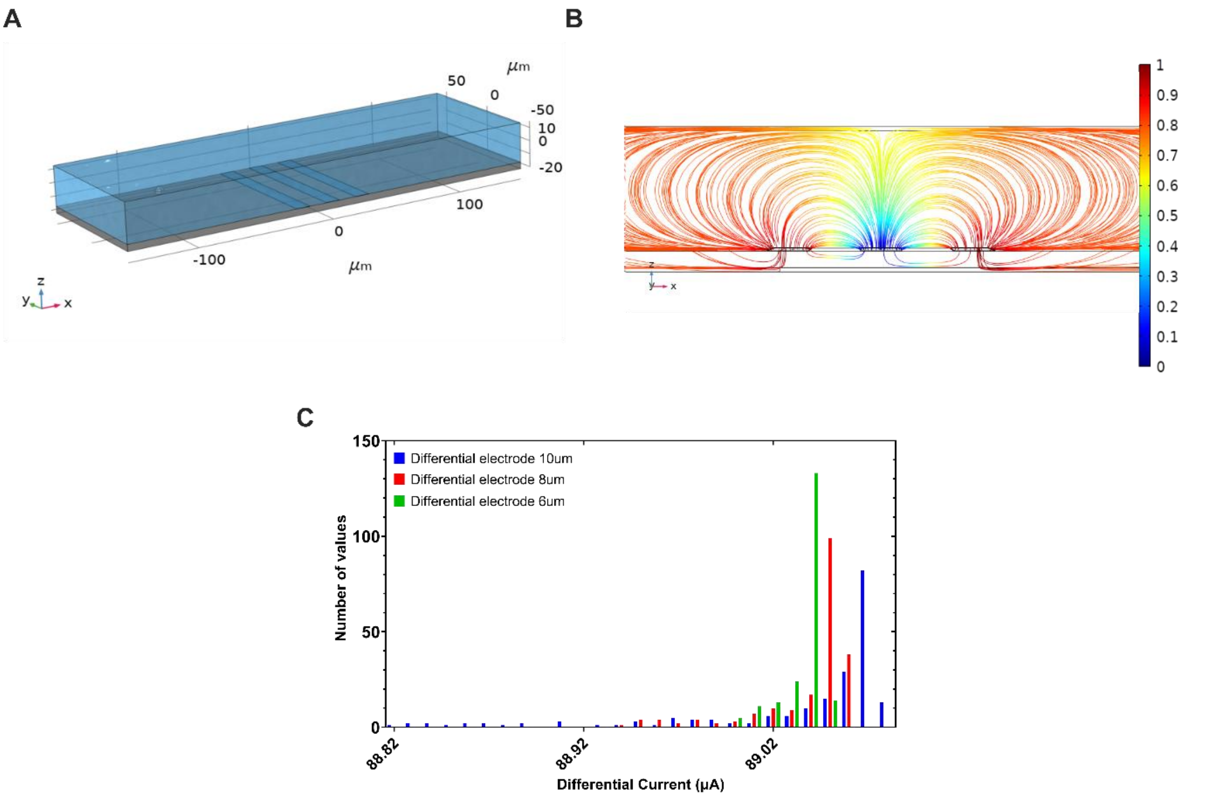

Figure S9 - Results of optimised three electrode differential coplanar device. (A) shows the geometry of the device, as modelled in COMSOL. (B) shows the electric field distribution within the sensing region of the device, while (C) shows the accuracy of the device by plotting the variation in magnitude of the signal for varying cell sizes (6µm, 8µm and 10µm, respectively). It can be seen in (C) that even the optimised three electrode differential coplanar device does not accurately resolve between cell population and therefore would be entirely reliant on additional analysis techniques in order to accurately size these cells.

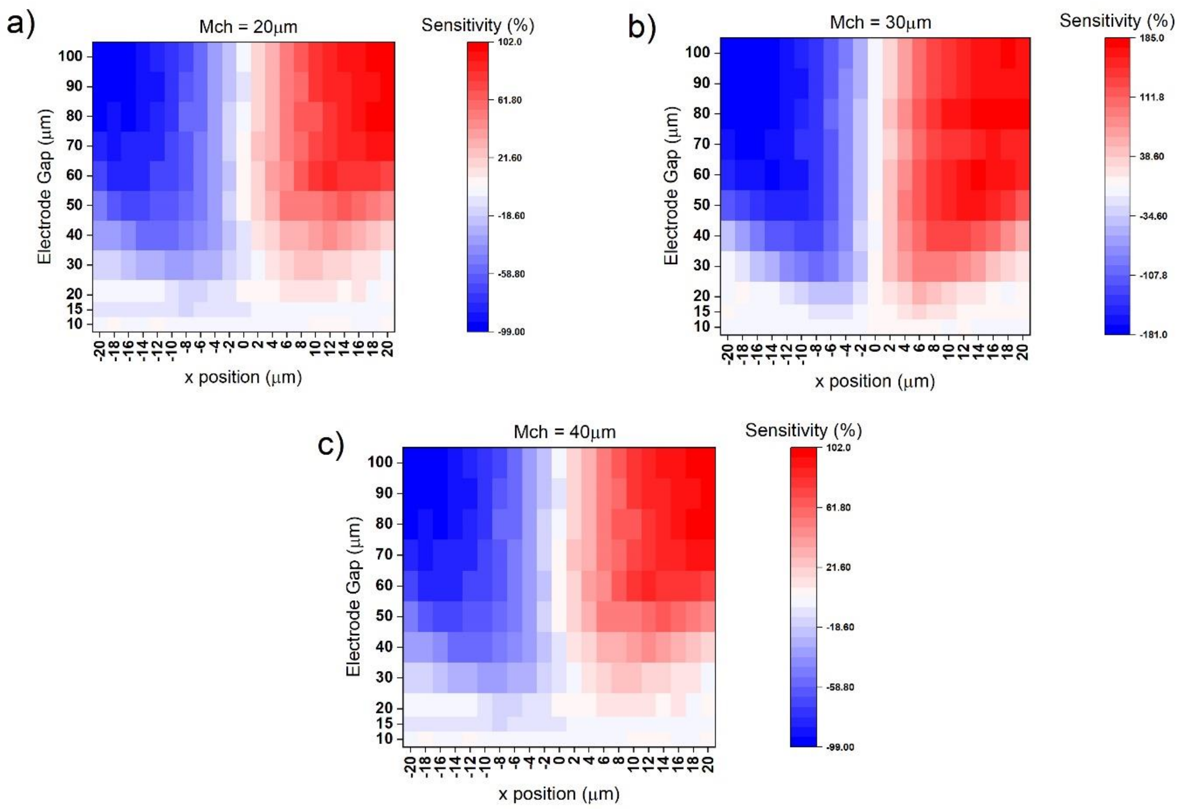

Figure S10 - The heat maps of this figure show the results of studies which were carried out to determine the peak sensitivity for three electrode differential sandwich devices with various configurations. (a) shows the device sensitivity mapped against the simulated variations in the electrode gap for a three electrode differential sandwich device with a microfluidic channel of 20μm diameter. (b) shows the device sensitivity mapped against the simulated variations in the electrode gap for a three electrode differential sandwich device with a microfluidic channel of 30μm diameter. (c) shows the device sensitivity mapped against the simulated variations in the electrode gap for a three electrode differential sandwich device with a microfluidic channel of 40μm diameter

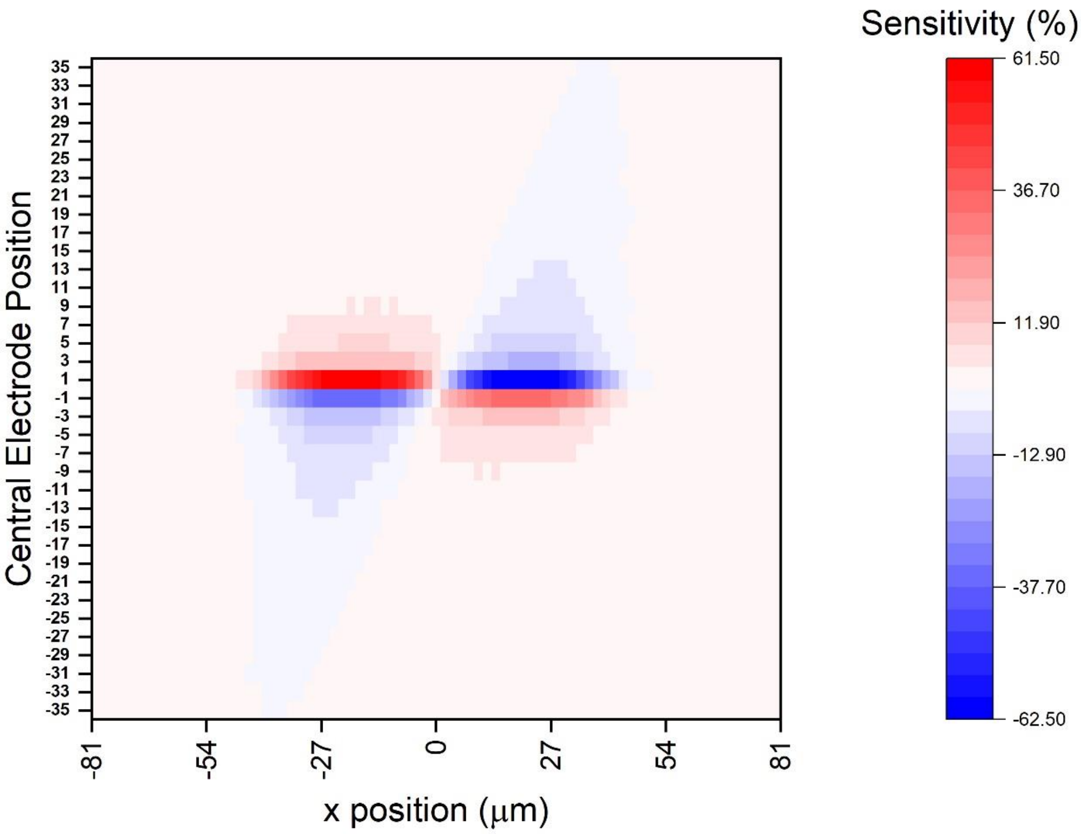

Figure S11 - The heat map shows the sensitivity of the three electrode, differential sandwich device for numerous configurations of cell position within the device and variation of the central electrode. This study was carried out in order to confirm assumptions that the peak sensitivity for this device was obtained with the central electrode located directly between the outer excitation and sensing electrodes.

References

1. Mei, Z., Liu, Z. & Zhou, Z. A compact and low cost microfluidic cell impedance detection system. *AIMS Biophys.* **3**, 596–608 (2016).
2. Hayashi, Y. & Asami, K. Dielectric Properties of Blood and Blood Components. *Dielectr. Relax. Biol. Syst.* 363–387 (2015) doi:10.1093/acprof:oso/9780199686513.003.0014.
3. Mata, A., Fleischman, A. J. & Roy, S. Characterization of Polydimethylsiloxane (PDMS) Properties for Biomedical Micro/Nanosystems. *Biomed. Microdevices* **7**, 281–293 (2005).
4. Nag, A., Feng, S., Afsarimanesh, N., Mukhopadhyay, S. & Kosel, J. Development of Novel Gold/PDMS Sensors for Medical Applications. *Int. Symp. Med. Inf. Commun. Technol. ISMICT* **2018-March**, (2018).
5. Hasan, M., Zhao, J. & Jiang, Z. A review of modern advancements in micro drilling techniques. *J. Manuf. Process.* **29**, 343–375 (2017).
